# Stimulation of TRPV1 by Green Laser Light

**DOI:** 10.1155/2012/857123

**Published:** 2012-12-17

**Authors:** Quanbao Gu, Lina Wang, Fang Huang, Wolfgang Schwarz

**Affiliations:** ^1^Shanghai Research Center for Acupuncture and Meridians, 199 Guoshoujing Road, Shanghai 201203, China; ^2^Acupuncture and Moxibution College, Shanghai University of Traditional Chinese Medicine, 1200 Cailun Road, Shanghai 201203, China; ^3^State Key Laboratory of Medical Neurobiology, Shanghai Medical College, Fudan University, Shanghai 200031, China; ^4^Institute for Biophysics, J. W. Goethe University, Max-von-Laue Stra**β**e 1, 60438 Frankfurt am Main, Germany

## Abstract

Low-level laser irradiation of visible light had been introduced as a medical treatment already more than 40 years ago, but its medical application still remains controversial. Laser stimulation of acupuncture points has also been introduced, and mast-cells degranulation has been suggested. Activation of TRPV ion channels may be involved in the degranulation. Here, we investigated whether TRPV1 could serve as candidate for laser-induced mast cell activation. Activation of TRPV1 by capsaicin resulted in degranulation. To investigate the effect of laser irradiation on TRPV1, we used the *Xenopus* oocyte as expression and model system. We show that TRPV1 can functionally be expressed in the oocyte by (a) activation by capsaicin (*K*_1/2_ = 1.1 **μ**M), (b) activation by temperatures exceeding 42°C, (c) activation by reduced pH (from 7.4 to 6.2), and (d) inhibition by ruthenium red. Red (637 nm) as well as blue (406 nm) light neither affected membrane currents in oocytes nor did it modulate capsaicin-induced current. In contrast, green laser light (532 nm) produced power-dependent activation of TRPV1. In conclusion, we could show that green light is effective at the cellular level to activate TRPV1. To which extend green light is of medical relevance needs further investigation.

## 1. Introduction

Low-level laser irradiation in the mW/cm^2^ range of visible and near-infrared (NIR) light had been introduced as a medical treatment already in the late 60s (see [1]), but its medical application still remains controversial. One major reason is that the cellular and molecular photo/biological responses are highly complex.

Since light below 600 nm is strongly absorbed in tissue by haemoglobin and melanin, and above 1200 nm by the water, medical application focused on the red and NIR range (see e.g., [2]). As possible photoreceptor cytochrome oxidase C has been suggested [3, 4]. Also in a modern variant of Chinese medicine, laser light is used to stimulate acupuncture points (see e.g., [5]). In traditional Chinese medicine, acupuncture points are stimulated by the needling procedure; it could be demonstrated that this leads to the degranulation of mast cells, which forms an essential early step in acupuncture-induced analgesia [6]. The degranulation in acupuncture points cannot only be induced by the mechanical stress during needle manipulation or osmotic stress, but also by high temperatures like those that are applied during moxibustion. Even irradiation of mast cells with red laser light, that is used in the laser acupuncture [5, 7], results in degranulation [8]. More recently, irradiation of acupoints with blue laser light has been introduced in medical treatment [9].

In the work by Zhang et al. [8], it was shown that the degranulation induced by mechanical stress, high temperature, and red light involves activation of TRPV2, a member of the family of transient-receptor-potential (TRP) ion channels. Application of blue laser light was also demonstrated to elicit mast-cell degranulation, and the involvement of another TRP channel, TRPV4, was suggested [10]. In addition to these two TRPV channels, mast cells also express TRPV1 [8]. In our work presented here we investigated to which extent TRPV1 can be activated by blue, green, and red laser light.

To investigate effects of laser light on TRPV1 and to avoid interference with the other members of TRPV family in the mast cells, we used *Xenopus* oocytes as a model system with heterologously expressed TRPV1. Activity of TRPV1 was monitored under voltage clamp as TRPV1-mediated current.

## 2. Materials and Methods

### 2.1. Transcription of TRPV1 cDNA

Full length human TRPV1 cDNA was cut from the vector pCAGGSM2-IRES-GFP-R1R2/TrpV1 (kindly provided by Dr. B. Nilius, University of Leuven, Belgium) with restriction enzyme Cla I and EcoR I and subcloned into the in vitro transcription vector pTLN digested with the same enzymes. The recombinant plasmid named pTLN-hTRPV1 was confirmed by DNA sequencing. 5 *μ*g pTLN-hTRPV1 were linearized by Hpa I and purified. The in vitro transcription was carried out using 2 *μ*g linearized pTLN-hTRPV1 under the guideline of SP6 transcription kit (Ambion). TRPV1 cRNA was stored at −20°C for the following experiments. 

### 2.2. Cell Culture

Human mast cells HMC-1 (kindly provided by Dr. J. H. Butterfield, Mayo Clinic, USA) were cultured in IMDM (Gibco, Invitrogen, USA), supplemented with 2 mM L-glutamine, 25 mM HEPES, 10% (v/v) fetal bovine serum (Gibco, Invitrogen), and 1% penicillin and streptomycin (Gibco, Invitrogen, USA) in a 95% humidity-controlled incubator with 5% CO_2_ at 37°C. 

### 2.3. Expression of TRPV1 Protein in *Xenopus* Oocytes

To investigate effects of laser irradiation on the TRPV1 protein, we used the *Xenopus* oocyte for heterologous expression and applied voltage-clamp techniques. Females of the clawed toad *Xenopus laevis* (Maosheng Bio-Technology Co., Shanghai, China) were anaesthetized with tricaine (1 g/L H_2_O, MS222, Sandoz, Basel, Switzerland) or in ice water. Parts of the ovary were removed and treated with 0.3 units per mL liberase (Roche) for 3 h to remove enveloping tissue and to obtain isolated oocytes. For expression of TRPV1 protein, oocytes of stage V or VI [11] were selected and injected with 20 ng cRNA (at 1 ng/1 nL) two to three days before the experiments; uninjected oocytes served as controls. The cells were stored at 19°C in oocyte Ringer's-like solution (G-ORi, see Section 2.6). Experiments were performed at room temperature (24–26°C).

### 2.4. Voltage-Clamp Experiments

We applied conventional two-electrode voltage clamp using Turbo TEC-03 with CellWorks software (NPI electronic, Tamm, Germany) to measure membrane currents. To determine steady-state current-voltage dependencies (IV curves), membrane currents were averaged during the last 20 ms of 200 ms, rectangular voltage pulses from −150 to +30 mV in 10 mV increments; the pulses were applied from a holding potential of −60 mV. To avoid changes at the reference bath electrode due to changes in Cl^−^ activity, the electrode was uncoupled from the bath via an ORi-filled channel. 

### 2.5. Laser Stimulation

For laser stimulation of the oocytes continuous-wave (CW) lasers were used, for 406 nm the CW Laser 1051390/AF (COHERENT), for 532 nm the CW Laser SUWTECH LDC 1500 (Shanghai Uniwave Technology Co., Ldt), and for 657 nm the CW Laser SB2007047 (Shanghai University of Traditional Chinese Medicine). Fibre optics were used to guide the laser light close to the oocyte (5 mm) with output powers of 5 mW, 36 mW, and 5–40 mW—for the blue, red, and green laser light, respectively. The spot size at the position to the oocyte was 2 mm in diameter, oocytes had a diameter of 1–1.2 mm. In several experiments using a one-millimetre thermoprobe, we confirmed that the applied laser light could not produce any significant change in temperature; at most an increase of 0.5 degrees was detectable after 30 min of irradiation. Taking into account that the oocyte was in addition continuously superfused with fresh solution of room temperature, effects of changing temperature can be excluded.

### 2.6. Solutions

Standard ORi (Oocyte Ringer's) solution contained (in mM) 90 NaCl, 2 KCl, 2 CaCl_2_, and 5 MOPS (pH 7.4, adjusted with Tris). For incubation of the oocytes, the ORi was supplemented with 70 mg/L Gentamycin (G-ORi). Stock solutions of capsaicin (1 mM) were prepared in ethanol and of ruthenium red (RuR, 6 mM) in distilled water. The bath solution for HMC-1 cells contained (in mM) 150 NaCl, 5 KCl, 2 CaCl_2_, 5 MgCl_2_, 4 D-sorbitol, and 10 HEPES (pH 7.4 adjusted with NaOH).

### 2.7. Data Analysis

For judging statistical significant effects of laser irradiation on current-voltage dependencies, *t*-tests were performed for at least 2 different potentials (−100 and −50 mV). Mean values were considered as statistically different on the basis of *t*,  *P* < 0.03.

## 3. Results

### 3.1. Functional Expression of TRPV1 in the Oocytes

To demonstrate that TRPV1 was functionally expressed in the oocytes, several specific characteristics of TRPV1-mediated current were investigated.

**Figure 1:**
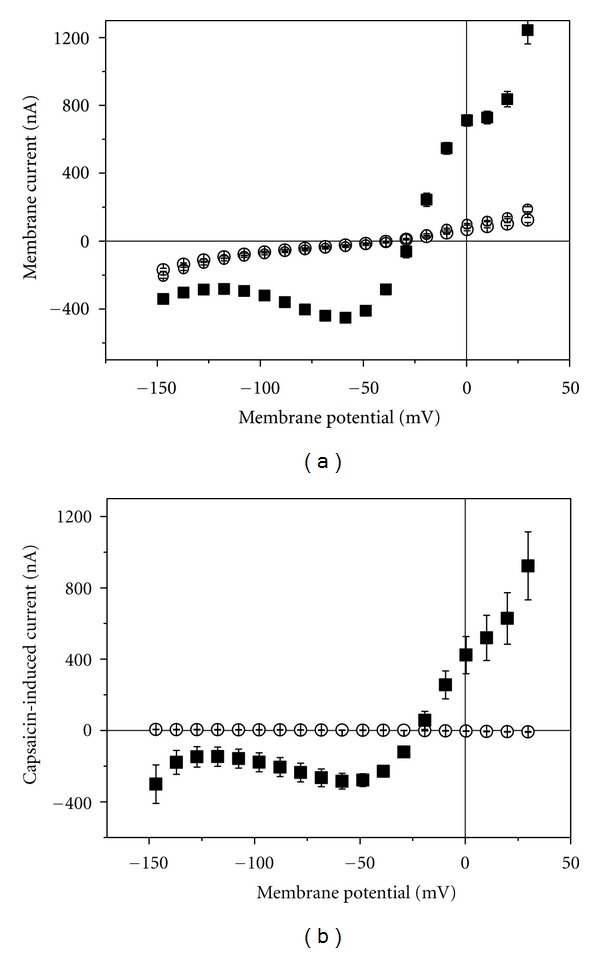
Effect of capsaicin application on current-voltage curves. (a) Membrane current in oocytes injected with TRPV1-cRNA. Large open circles before, filled squares during, and small open circles after application of 500 nM capsaicin. (b) Capsaicine-induced current in uninjected oocytes (open circles) and cRNA-injected oocytes (filled squares). All data are averages from 8 experiments (±SEM).

TRPV1 is known to be activated by capsaicin [12, 13]. Oocytes injected with cRNA for TRPV1 responded to application of 500 nM capsaicin with an increase in membrane current that completely disappeared after washout (Figure 1(a)). To correct for possible drift with time, capsaicin-induced current *I*_cap_ was calculated according to

(1) Icap=I1before−I1after2−I2,

where *I*1 is the current in the absence of capsaicin (before or after the application of the agonist) and *I*2 the current in the presence. The difference current will be considered as current mediated by TRPV1 (Figure 1(b)); oocytes not injected with cRNA, never exhibited any capsaicin-sensitive current (Figure 1(b)). The current-voltage dependence is characterised by outward rectification. Oocytes not injected with cRNA exhibited no response to capsaicin.

**Figure 2:**
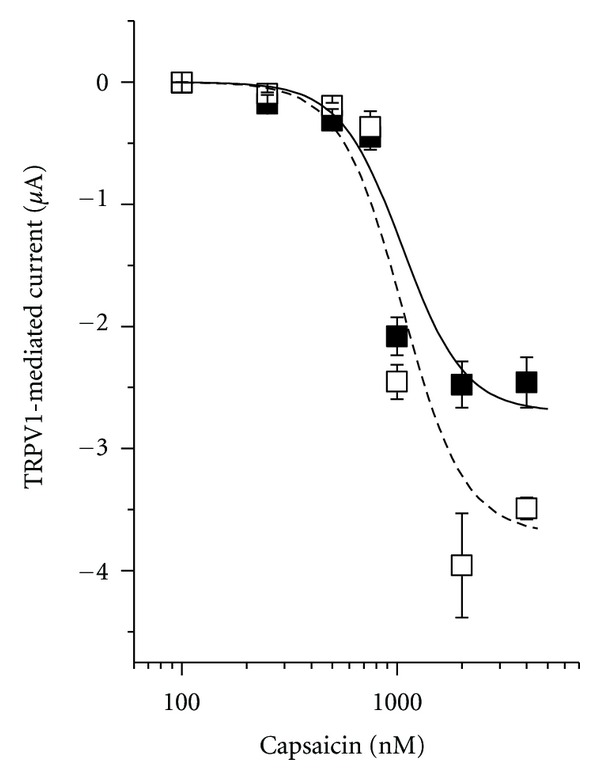
Dependence of TRPV1-mediated current on capsaicin concentration. Filled squares current at −60 mV and open squares at −100 mV. Data are averages from 7 experiments (±SEM). Lines represent approximations of the concentration dependencies with the same *K*_1/2_ value of 1.06 *μ*M.

The dependence of the TRPV1-mediated current showed strong dependence on capsaicin concentration (Figure 2) and can be approximated by

(2) I=Imax⁡[capsaicin]3[capsaicin]3+K1/23,

with an *K*_1/2_ value of 1.06 *μ*M for −60 as well as −100 mV.

**Figure 3:**
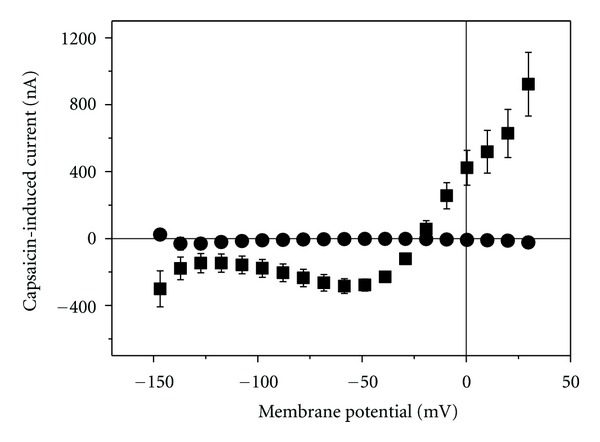
Inhibition of capsaicin-induced current by RuR. Filled square current-voltage dependence of current induced by 500 *μ*M capsaicin, filled circles in the simultaneous presence of 12 *μ*M RuR. Data are averages (±SEM) of 14 measurements in the absence and of 4 measurements in the presence of RuR.

Another characteristic of TRPV1 is its inhibition by ruthenium red (RuR) [14].  Figure 3 shows that 12 *μ*M RuR completely blocked the capsaicin-induced current in the oocytes over the entire potential range. In control oocytes not injected with cRNA, no RuR-sensitive current component could be detected.

**Figure 4:**
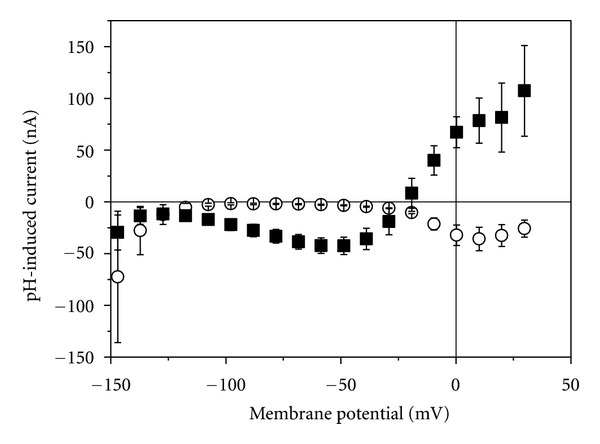
Effect of pH reduction on membrane current. Change current in response to a reduction of external pH from 7.4 to 6.2 in noninjected oocytes (open circles) and in cRNA-injected cells (filled squares). Data for injected cells are averages (±SEM) of 7 measurement; for the control cells 2 measurements were performed.

TRPV1 can also be activated by reduced pH [12]. Over the potential range of −40 to −120 mV, reducing the extracellular pH hardly affected the membrane current in noninjected oocytes (Figure 4). In cRNA-injected oocytes, the change in pH from 7.4 to 6.2 activated a current (Figure 4) with similar voltage dependence as the capsaicin-induced current (compare Figure 1(b)).

**Figure 5:**
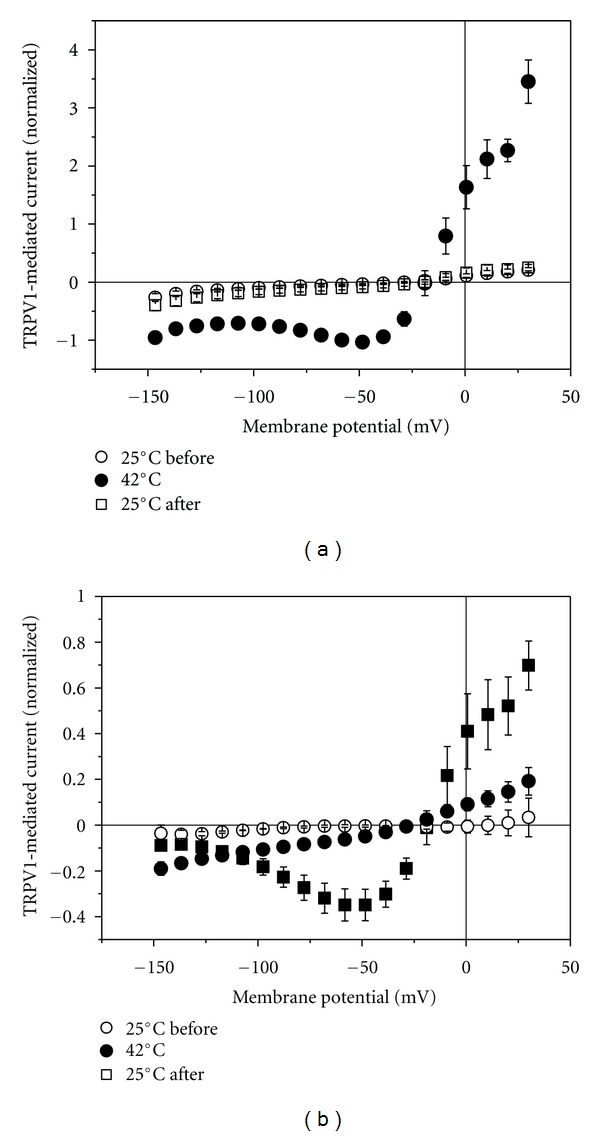
Effect of elevated temperature on membrane current of TRPV1-expressing oocytes. (a) Effect of 42°C (filled circles) in comparison to 25°C before and after the raise in temperature. (b) Effect of 35°C (filled circles) in comparison to 25°C before the raise in temperature and to the current induced by 500 nM capsaicin (filled squares).

Physiologically TRPV1 functions as thermosensor responding to noxious temperatures exceeding 42°C. If the temperature of the solution superfusing the oocyte was increased from room temperature of 25°C to 42°C, outward-rectifier current, typical for the capsaicin-induced current, was stimulated (Figure 5(a)). This increase in current was completely reversible when the temperature was returned to 25°C. In the presence of RuR, no such current could be activated at 42°C (not illustrated). Also at 35°C only a tiny current component became apparent with voltage-dependence different from TRPV1-mediated current (Figure 5(b)).

In the following we investigated to which extent TRPV1 function can be modulated by laser light of three different wave lengths: red light of 637 nm, blue light of 406 nm, and green light of 532 nm. In oocytes not expressing TRPV1 with none of the wavelengths, any current modulation was detectable, even at the highest output powers of 40 mW.

### 3.2. Effect of Red Laser Light on TRPV1-Mediated Current

**Figure 6:**
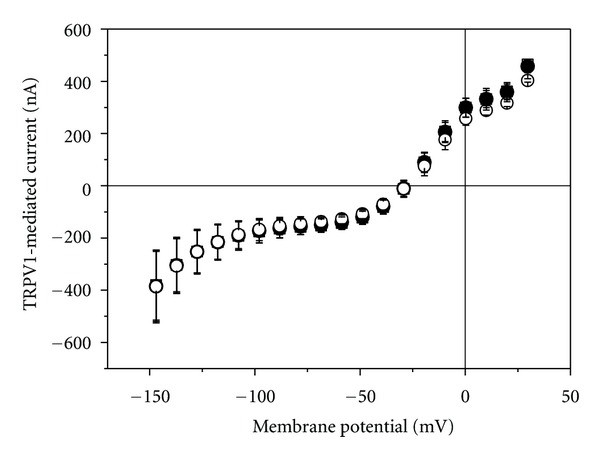
Effect of red laser light on current-voltage curve of TRPV1-mediated current. TRPV1 was activated by 500 nM capsaicin. Open square were obtained before irradiation, filled circles at the end of a 2 min lasting irradiation period (637 nm, 36 mW), and open circles 2 min after the laser light was turned off. Data represent averages from 3 oocytes (±SEM).

In medical low-level laser application, including laser acupuncture, red laser light is often applied. In the absence of capsaicin, no change in membrane current was detectable when red laser light (637 nm, 5 mW) was applied (not shown); even at 36 mW no sign of light-induced current was visible (for holding current at −60 mV see Figure 9). Also when TRPV1 was activated by 500 nM capsaicin, a 2 min time period of irradiation could not be significantly modulated TRPV1-mediated current (Figure 6).

### 3.3. Effect of Blue Laser Light on TRPV1-Mediated Current

**Figure 7:**
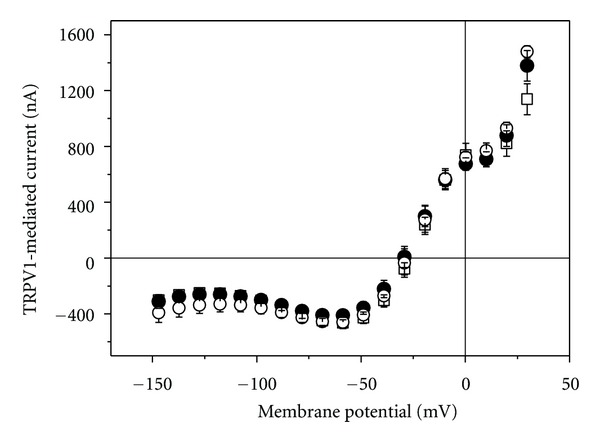
Effect of blue laser light on current-voltage curve of TRPV1-mediated current. TRPV1 was activated by 500 nM capsaicin. Open square were obtained before irradiation, filled circles at the end of a 2-min lasting irradiation period (406 nm, 5 mW), and open circles 2 min after the laser light was turned off. Data represent averages from 3 oocytes (±SEM).

Recently, low-level blue laser light was introduced in laser acupuncture [9]. Therefore, we also tested for the effect of 406 nm on TRPV1-mediated current. Similarly to the red laser light, an output power of 5 mW could not affect membrane current (for holding current at −60 mV see Figure 9). Also when TRPV1 was activated by 500 nM capsaicin, a 2 min time period of irradiation could not significantly modulate TRPV1-mediated current (Figure 7). 

### 3.4. Effect of Green Laser Light on TRPV1-Mediated Current

**Figure 8:**
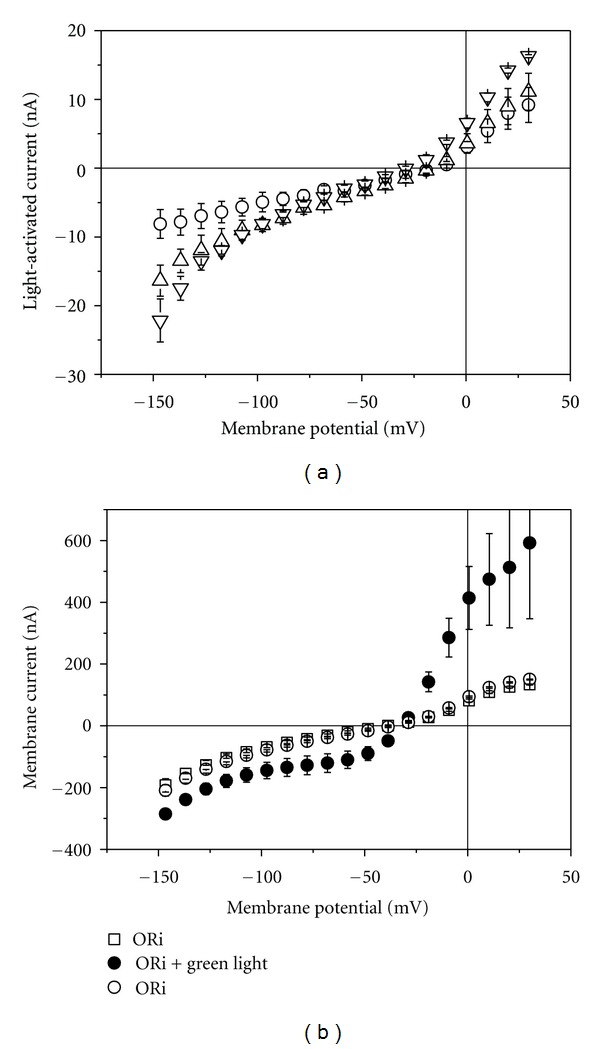
Effect of green laser light on current-voltage curve of TRPV1-mediated current. (a) Light-activated current in oocytes injected with cRNA for TRPV1; output power 5 mW (circles), 10 mW (triangle up), and 20 mW (triangles down). Data represent averages from 3 oocytes (±SEM). (b) Membrane current before (open squares), during (2 min after light was turned on, filled circles), and after irradiation at 40 mW. Data represent averages from 5 oocytes (±SEM).

**Figure 9:**
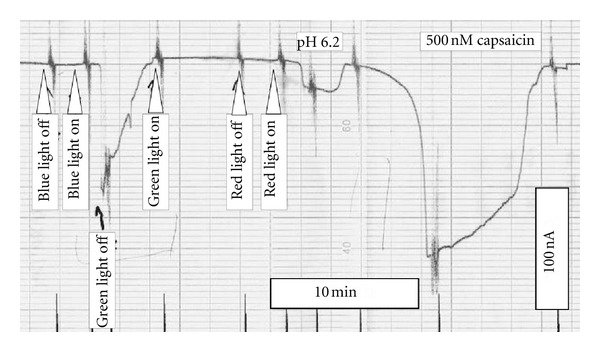
Pen recording of holding current at −60 mV. Downward deflexions represent activation of inward current. Red light of 637 nm was applied at 36 mW, green light of 532 nm at 40 mW, and blue light of 406 nm at 5 mW.

In contrast to the red and blue laser light, green laser light (532 nm) activated in cRNA-injected oocytes even in the absence of capsaicin a current that increased with increasing output power (Figure 8(a)). Already at 5 mW a significant TRPV1-mediated current could be activated. The effect gradually increased with time reaching a maximum steady-state current within 2 min of irradiation (for 40 mW compare Figure 9). When the laser light was turned off, the current instantaneously dropped to the same level as before irradiation. At laser output power of 40 mW, the typical outward rectification of TRPV1 channel was clearly apparent (Figure 8(b)).

### 3.5. Capsaicin Induces Degranulation in HMC-1

**Figure 10:**
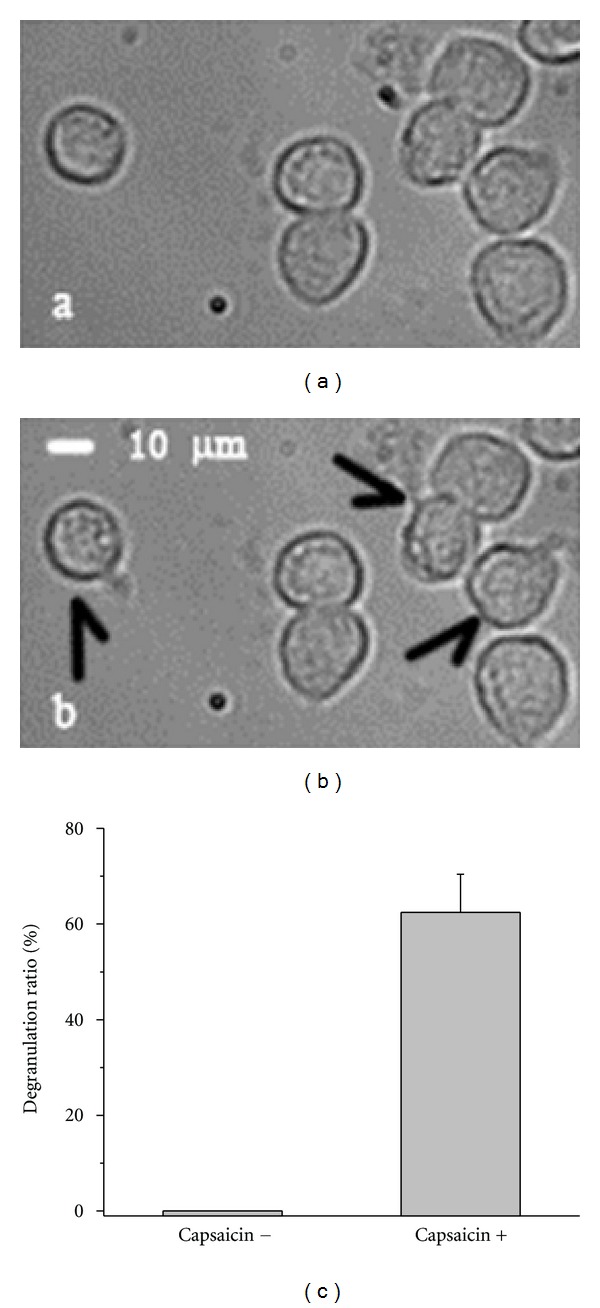
Degranulation of human mast cells induced by capsaicin. (a) Human mast cells HMC-1 incubated in bath solution in the absence of the TRPV1-specific agonist capsaicin. (b) HMC-1 cells having been superfused for 5 min with bath solution containing 1 *μ*M capsaicin. The arrows point to the degranulating cell. (c) Percentage of degranulated HMC-1 cells in the absence and presence of 500 nM capsaicin (exposition time 10 min, values present averages of 3 independent experiments ±SEM).

The experiments described above were done on the model system *Xenopus* oocyte. To support the idea that mast cell degranulation and activation of TRPV1 might be involved in acupuncture effects, we tested whether TRPV1 activation can induce mast cell degranulation. Figures 10(a) and 10(b) illustrate that application of 1 *μ*M capsaicin (about *K*_1/2_ value for TRPV1 activation, see Figure 2) indeed led to degranulation. Already at 500 nM clear activation of TRPV1 was possible (Figure 1); after application of 500 nM capsaicin for 5 min 62 ± 8% of the cells had degranulated (Figure 10(c)).

## 4. Discussion

### 4.1. TRPV1 Is Functionally Expressed in the Oocytes

We have demonstrated that TRPV1 was functionally expressed in the oocytes by showing TRPV1-specific characteristics. Oocytes injected with the cRNA for TRPV1 exhibited capsaicin-inducible current (Figure 1) with a *K*_1/2_ value of about 1 *μ*M (Figure 2) which is similar to the value reported by others [12, 13]. Also the current-voltage dependence with outward-rectifier characteristic was reported for TRPV1-mediated current [14, 15]. The inhibition by RuR (Figure 3) and activation by reduced pH (Figure 4) of such outward-rectifying current are additional indications [12, 14] that TRPV1 was functionally expressed in the oocytes.

Within the TRPV family, TRPV1 is characterized also by temperature sensitivity with activation by temperature above 42°C [16, 17]. Increasing the temperature even to 35°C only slightly increased the membrane conductance (Figure 5(a)), but as soon as the temperature reached about 42°C, current was induced with the outward-rectifying current-voltage dependence typical for TRPV1 (Figure 5(b)).

### 4.2. TRPV1 as a Candidate for Laser-Induced Degranulation

It was demonstrated previously that degranulation of mast cell is an essential initial step in acupuncture-induced pain relief [6]. In addition, it was demonstrated that various physical stimuli used in Chinese Medical Treatment can produce mast-cell degranulation [8]. These stimuli include mechanical stress, which is applied during the acupuncture needle manipulation [18–20] via the connective tissue to the mast cells, and high temperatures, which are applied during moxibustion to the acupuncture points [21]. Also red laser light in the 630 to 640 nm range is often applied to acupuncture points [7, 9, 22]. All three physical stimuli can elicit mast-cell degranulation that involves activation of TRPV2 ion channels [8].

Because of the optical window of tissue in the range of 600 to 1200 nm, red and NIR light have a penetration depth of up to several mm [23] and have mainly been used to investigate photo biomodulation and stimulation including its application in laser acupuncture. Despite the fact that blue light has a penetration depth in only the sub-mm range, nevertheless, irradiation of acupuncture points by blue laser light was introduced into laser acupuncture recently [24]. Irradiation of mast cells with a 405 nm laser indeed leads to mast-cell degranulation that was attributed to an intracellular increase of Ca^2+^ by activation of TRPV4 [10]; TRPV4 is also expressed in mast cells [8, 10].

While red and blue laser light seem to stimulate activation of TRPV2 and TRPV4, respectively, these wave lengths were ineffective for TRPV1 (Figures 6 and 7). On the other hand, for TRPV1 green laser light was a very effective stimulus (Figure 8). At 40 mW, the light induced current amounted to about 80 nA (at −60 mV, see Figure 9), which is comparable to the 85 nA of current that can be activated by 250 nM capsaicin (Figure 2). 

Photo biostimulation particularly in the red and NIR range have been attributed to absorption by cytochrome oxidase C. Interestingly, the stimulation of TRPV1 by the green light is dose-dependent, but after about 2 min a steady state is reached, and when turning off the light the TRPV1-mediated current instantaneously vanishes. The physical basis for this is currently under investigation.

In our work, we focused on laser-light-induced mast-cell degranulation that might form an initial step in acupuncture induced analgesia. Detailed investigations have elucidated that irradiation of peripheral nerve with red or NIR laser light may also be of relevance for analgesic effects (for a detailed review see [25]). In particular, decreased conduction velocities have been reported in experiments on humans and animals with involvement of A*δ* and C fibres. In our experiments, we could demonstrate the effectiveness of green laser light on TRPV1. Interestingly, TRPV1 is highly expressed in peripheral A*δ* and C fibres [12], and hence, may also form in the nerve cells a target for green laser-light stimulation. It was reported that within acupuncture points, TRPV1 shows higher expression on A*δ* and C fibres than in nonacupuncture points and this becomes even increased after electroacupuncture [26]. The authors conclude that the higher expression of TRPV1 and its upregulation in response to acupuncture may be involved in the transmission of the acupuncture signal.

In addition to the results in our model system *Xenopus* oocyte, we could show that activation of TRPV1 by the specific agonist capsaicin can indeed induce degranulation (Figure 10). We, therefore, like to suggest that in addition to red and blue laser light also green light with its intermediate penetration depth might be a useful tool in medical treatment. 

## Abbreviations

CW laser: Continuous-wave laser
ORi: Oocyte Ringer's
RuR: Ruthenium red
TRP: Transient receptor potential
TRPV1: Transient receptor potential valinoid-sensitive, isoform 1.
